# Development of rapid guidelines: 2. A qualitative study with WHO guideline developers

**DOI:** 10.1186/s12961-018-0329-6

**Published:** 2018-07-13

**Authors:** Ivan D. Florez, Rebecca L. Morgan, Maicon Falavigna, Sérgio C. Kowalski, Yuan Zhang, Itziar Etxeandia-Ikobaltzeta, Nancy Santesso, Wojtek Wiercioch, Holger J. Schünemann

**Affiliations:** 1 Department of Health Research Methods, Evidence and Impact and Mac GRADE Center, McMaster University Health Sciences Centre, Room 2C16, 1280 Main Street West, Hamilton, ON L8N 4K1 Canada; 2 Department of Pediatrics, University of Antioquia, Cra. 51D #62-29, Medellin, 050001 Colombia; 3 Hospital Moinhos de Vento, Porto Alegre, Brazil; 4 National Institute of Science and Technology for Health Technology Assessment, Universidade Federal do Rio Grande do Sul, Porto Alegre, Brazil; 5 Department of Internal Medicine, Division of Rheumatology, Universidade Federal do Paraná, R. Gen. Carneiro, 181, Curitiba, PR Brazil; 6 Department of Medicine, McMaster University, Health Sciences Centre, Room 2C14, 1280 Main Street West, Hamilton, ON L8S 4K1 Canada; 7 Cochrane Canada Center, McMaster University, Health Sciences Centre, Room 2C14, 1280 Main Street West, Hamilton, ON L8S 4K1 Canada

**Keywords:** Guideline, Emergencies, Methodology, Rapid reviews, Guideline development, Clinical guidelines

## Abstract

**Background:**

Situations such as public health emergencies and outbreaks necessitate the development and publication of high-quality recommendations within a condensed timeframe. For example, WHO has produced examples of and guidance for the development of rapid guidelines (RGs). However, more information is needed to understand the experiences and perceptions of guideline developers. This is the second of a series of three articles addressing methodological issues around RGs. This study describes the perceptions and experiences of guideline developers at WHO about RGs.

**Methods:**

We conducted interviews consisting of open- and closed-ended questions with guideline developers at WHO. Our analysis described the definition and rationale of RGs, the differences from regular guidelines with regard to timelines from topic definition until publication, barriers to identifying the evidence and the lack of a standard methodology to develop RGs.

**Results:**

We interviewed 10 participants, the majority of whom were comfortable with the current WHO definition of RGs. Most stated that the rationale for developing RGs should be in response to new evidence about efficacy, cost-effectiveness or safety. Respondents differed with regards to the amount of time RGs should take. While the majority of participants agreed that guidelines should be based on a systematic review, this step in the process was considered the most time and resource intensive. Challenges for developing RGs included limited personnel and financial resources as well as the lack of evidence. Facilitators, in turn, that may improve RG development include additional financial and personnel resources as well as the use of virtual meetings.

**Conclusions:**

While our study suggests a strong need and rationale for the development of RGs, standardisation of timelines and guidance on panel composition, peer-review process, conduct of meetings and sources of permissible evidence require further research.

**Electronic supplementary material:**

The online version of this article (10.1186/s12961-018-0329-6) contains supplementary material, which is available to authorized users.

## Background

Healthcare guidelines are recommendations developed to inform decision-making among healthcare providers and consumers, as well as other stakeholders [1]. A variety of organisations, including professional and scientific associations, academic institutions and governmental organisations, develop guidelines.

High-quality guidelines are resource intense, requiring both human and economic resources. The time needed to develop practice guidelines can range from 18 to 24 months [2, 3]; however, the timeframe is dependent on several factors, including the number of questions and the scope of the guideline [4].

In 2006, a review of WHO’s approach to guideline development and use of evidence revealed shortcomings that, in turn, led to organisational changes of its guideline development efforts [5, 6]. This included the creation of a detailed handbook for guideline development according to best practice for guideline development and a committee overseeing the efforts for the organisation. The various editions of the handbook provide guidance in planning, developing and publishing a guideline that meets WHO standards [7]. The latest edition of the WHO Handbook recommends that guidelines are developed over 6–24 months [4].

However, in response to specific situations (e.g. outbreaks, public health emergencies, etc.), there is an urgent need for expedited guidance to inform decision-making. The term ‘rapid advice guidelines’ has been used by WHO to define guidelines that must be developed in a very short period of time in response to specific urgent healthcare scenarios [4]. When rapid advice guidelines are needed, WHO suggests that the timeframe to develop a recommendation should be 1–3 months. Although this is a very short period of time, rapid guidelines (RGs) should still be developed with adequate quality standards, which is a real challenge for guideline developers. As WHO has developed and issued multiple rapid advice guidelines on clinical and public health topics, members of the organisation were contacted to share their knowledge, attitudes and beliefs regarding the RG development process [8, 9].

This article is the second in a series of three evaluating the state, perceived challenges and solutions related to RG development. Based on a systematic survey, the first article in this series described the concept and importance of RGs in decision-making and provided a number of examples including an assessment of their quality [10]. This article describes current perceptions and experiences, as well as the perceived barriers and facilitators for the development of RGs based on semi-structured interviews of guideline developers at WHO.

## Methods

This qualitative descriptive study describes the perceptions and experiences of guideline developers at WHO. This manuscript follows the recommendations provided by the Standards for Reporting Qualitative Research [11]. We used the term ‘rapid guidelines’ to refer to guidelines produced with a shortened timeframe to establish recommendations.

### Researchers’ characteristics

Four MD- and PhD-level researchers with experience in epidemiological methods conducted the interviews and performed the main analysis (IF, RM, MF and HLS). The researchers are methodologists and clinicians with experience in regular guidelines development and one of the researchers, and the senior author (HJS), has developed a RG for WHO that was used in some of the examples provided to interviewees (avian influenza RG).

### Participants and setting

We purposively sampled WHO staff who participated in rapid guidelines or whose work required rapid response. To gain a broad perspective of perceptions and experiences for this study, participants were selected from different departments throughout WHO and contacted directly based on their involvement with guideline development. We also intended to include a broad range of topic areas, such as acute infectious diseases, catastrophic events and chronic non-communicable diseases. A further selection criterion was to sample participants that have been working in the RG field. With the exception of one remote video interview, we conducted all interviews in person at WHO Headquarters in Geneva, Switzerland, in May 2013.

### Data collection tool and process

We utilised pilot-tested semi-structured interviews containing both closed- and open-ended questions to understand the process and development of RGs at WHO. The interviews had the main objective of capturing insights about the RG development process, to identify time consuming aspects of the standard process and potential shortcuts for guideline development, and to identify potential scenarios for RG development. We explored the experts’ current or previous experience with RG development and not the characteristics of the institution’s policies. Topics related to the RG development methods explored in the interviews were based on a previously conducted systematic review [10]. We considered the following key points/themes: the differences in timeline from topic definition until publication, the barriers to identify evidence, the peer-review process, and the lack of standard methodology to develop RGs. Two investigators developed an initial draft of interview questions (MF, HJS) and discussed it with other members of the research team to obtain consensus and finalise the questions. A pilot interview was conducted with a healthcare professional involved in the guidelines field. The research team approved a final set of 18 questions (Additional file 1).

A methodologist (MF) with a medical background conducted all interviews, which took 40 min on average and were conducted in English. Only the interviewer and the interviewee were present during the interview, except for one interview that was conducted with two interviewees at the same time and place upon the interviewees’ request. All interviews were recorded and were semi-structured, wherein the interviewer attempted to cover all questions and left the interviewees free space to develop their own answers. Closed-ended questions required responses based on a Likert scale (1 – strongly disagree, 7 – strongly agree). The interview began with a brief introduction about the objectives of the study, followed by a series of closed-ended questions related to the interviewee background (Additional file 1), their department in WHO, their current position and time in it, areas of interest and activity, academic background, clinical or public health background, formal education in research methods, participation in previous guideline development, and whether or not they had chaired or led any guideline panels. The second part of the instrument asked participants to read the WHO definition of rapid advice guidelines and answer a series of questions (Additional file 2). Finally, one example of the key steps that occurred during the development of WHO rapid advice guidelines and timelines (Additional file 3) were described. If the interviewee required clarification for any question, the interviewer provided neutral but informative responses.

Participants were ensured that the data would be treated confidentially, and that the results would be published without revealing the identities of the respondents. Informed consent was obtained from all participants. The research ethics review board at McMaster University reviewed and approved the study.

### Data management, analysis and synthesis

The raw data was recorded, transcribed, assessed to develop a thematic analysis, and coded into themes of similar meaning identified through the process of interviews and analysis. We established themes for coding, classified their meanings, and synthetised the statements when saturation was reached. We defined saturation as the absence of emergence of new ideas, concepts or suggestions. Two investigators (IF, RM) developed and independently applied the coding scheme to the qualitative data and discussed disagreements. We used a qualitative thematic analysis [12] to obtain main themes that summarise and describe the reported perceptions, opinions and thoughts of interviewees, based on the qualitative description method [13, 14].

## Results

Out of 12 invited experts, 10 individuals agreed to participate (two individuals refused to participate because of lack of availability during the time of data collection), and we conducted 9 interviews (two participants were interviewed together). Participant characteristics are described in Table 1 demonstrating different background areas, including malaria, tuberculosis, HIV, reproductive health, diabetes, and evidence-informed policy-making. Four interviewees were native English speakers and the remainder spoke English as a second language.

**Table 1:** Participant characteristics (*n* = 10)

Characteristics of participants interviewed	Number
Number of interviews	9
Participants with medicine background	10
Participants with public health background	8
Participants with formal training in research methods	5
Median number of guidelines each participant had participated up to the interview day	3.5 (range 0–8)

### Themes

#### WHO definition for rapid advice guidelines

The definition of WHO rapid advice guidelines (described in Additional file 2) was considered ‘adequate’ or ‘reasonable’ by participants. Some participants were concerned with the statement explaining ‘what is a public health emergency’. Others recommended replacing the term ‘interim‘ with ‘rapid’, and ‘guide’ or ‘guidance’ with ‘guideline’ because of the perception that ‘guideline’ can be interpreted as a systematic process that should include all the steps.

“*I would phrase it differently; ‘Emergencies with an impact in public health’, to make it broader: In the sense that… in all emergencies you will have intrinsically public health complements in it. Right? But some are caused by, you know, either manmade or not, but with more focus, like in pandemics, you have a more clear public health component*.”

### Additional potential or hypothetical situations for performing RGs

Emergence of new important evidence in terms of effectiveness, safety or cost-effectiveness about a new or old intervention was considered the most important reason to develop RGs (Table 2). Additional potential reasons mentioned were: “*Pressure from country members of the WHO*”, “*the need for giving an advice*”, or “*the need to respond to the public opinion*”. In response to a series of eight hypothetical scenarios, participants identified an emergent epidemic of an infectious disease and the management or control of biological, chemical or radioactive hazards as the most important reasons for developing RGs. Less agreement existed for scenarios describing infectious diseases due to multidrug-resistant organisms or new evidence about an infectious disease (Table 2).

**Table 2:** Circumstances to develop a Rapid Guideline

Scenarios	Median^a^	IQR^a^
Emergent epidemic of an infectious disease	7	7–7
Management/control of biological, chemical or radioactive hazards	7	6.2–7
Management of infectious diseases by MDR organisms in hospitals/ICU	5	4–6
New evidence: infectious diseases	6	6–7
New evidence: neglected diseases	6	5–6.7
Diagnostic tests	3	3–5
New evidence for chronic diseases	3	2–5.7
Post-traumatic stress disorder	4.5	2–6

*ICU* Intensive-care unit, *IQR* Interquartile range, *MDR* Multidrug-resistant

^a^*Agreement measured through a Likert scale from 1 to 7;* Strongly Agreed (7), Agreed (6), Somewhat Agreed (5), Neither Agreed or Disagreed (4), Somewhat Disagreed (3), or Disagreed (2), Strongly Disagreed (1)

#### Feasibility and obstacles

The most common perceived obstacle when developing RGs was the lack of personnel resources taking into account the shorter timeframe (i.e. selecting the work group and coordinator, and familiarising experts with the RGs process). Lack of evidence was perceived as an important obstacle and as a risk:

“*There may not be enough evidence and if something goes wrong, then WHO may be accused of either being guided by wrong evidence or having pharma industry pushing us to develop a guideline because there is only one medication available. That is the risk*.”

Lack of financial resources for the development and the implementation of RGs taking into account lack of availability of interventions, such as drugs, in some countries, were additional perceived obstacles.

#### Timeline and the most time-consuming aspects

Four participants described that the time should depend on the topic or disease, whereas another four considered 1–3 months as an adequate period but some flexibility would be required according to the topic. However, one participant clearly stated that the best period of time is less than 1 month. Three participants recommended the development of an ‘interim’ recommendation during the first week if it is needed, maintaining the 1- to 3-month period.

“*… what I would suggest as an alternative for this is to get a real rapid response stuff, that takes from 48 hours to 7 days… and which is extremely provisional, which has a big caution, and says: ‘this what we have, we are looking to see if there is something else…’*”

Four participants stated that evidence gathering and synthesis were the most time-consuming steps in the RG process.

“*Talking about the guideline, the most time-consuming process, of course is this process of evidence and gathering: the systematic review and meta/analysis, which is taking several months*”

One participant each responded that external review, drafting and editing the guideline, agreement among experts when there is not robust evidence, finding knowledgeable and available people in the specific topic, getting the guideline approval, and framing the right questions were the most time-consuming steps.

We specifically asked participants to read an example of the Avian Influenza RG (Additional file 3) and suggest improvements to the timeline. Although the Avian Influenza RG was developed in 3 months, publicising the RG after almost 5 months seemed long for two of the participants. Participants suggested that the time to completion may be shortened during panel composition and question formulation (*n* = 3), approval period (*n* = 2), and the writing of the text/formulation of the recommendation (*n* = 1). Other suggestions included (one participant each) meeting as soon as possible, a RG approval period of 48 h, involvement of the WHO Guideline Review Committee (GRC) in regular meetings (of note, the WHO GRC did not exist when the Influenza guideline was developed and approval processes at WHO were less structured), and to agree on the text of the recommendation during the meetings (although that was already part of the Avian Influenza RG process). Two participants suggested that the timeline could be met depending on other criteria, e.g. it would be “*feasible when we have a narrow scope*”. One participant stated that anticipation of the emergencies could allow for people to connect and begin the process earlier.

#### Systematic reviews and evidence synthesis

Four participants agreed on the importance of maintaining appropriate methods in the process: “*avoid shortcuts because of bias*”, “*not doing systematic reviews would lead to loss of data*” and “*rigor must be maintained*”. However, two participants recommended optimising the processes by making more efficient use of time and maintaining rigor, while others suggested avoiding some steps such as searching grey literature, restricting to the English language, or reducing the search parameters. Two participants insisted on the flexibility of the process based on the urgency of the situation, and two stated that systematic reviews should not be a routine (i.e. in cases of very urgent RGs the process and evidence should be given by experts, without systematic reviews and without methodologists).

### Panel composition

Three participants stated that all stakeholders should be included in the RGs development panel. One participant noted that including all stakeholders is important “*when we have limited evidence*”. Two participants recommended that panels might be smaller, while two recommended not including individuals with conflicts of interest.

“*Conflict of interest is a huge problem; If you consult experts, it gets out of control...*”

One participant explicitly stated that methodologists should not be part of the team, “*because of them, the process is slower and complicated*”.

“*Maybe we should not involve methodologists; experts would bring the best evidence, since they know what to present and the most important evidence…*”

Other participants suggested including health economists and one participant suggested balancing panels for gender and geographic location. Five participants would include patients while two participants would not do so. Six participants agreed that country member representatives should be included. Of those six, three specified that it would be “*important for implementation*”.

“*I think that if you are in an emergency situation where you really have to do rapid advice having a full representative panel is not always possible… I would try as much as possible to involve them* [country member representatives]*, but the process with the government to choose the appropriate participants is long and you do not always get the right people*”

### Additional criteria to include: costs and resources, values and preferences, and external review

Agreement of participants with regards to the need to include information on cost, values and preferences as well as on external peer-review of RGs is shown in Table 3. One participant stated that costs must be taken into account at the implementation step. Two described that information about costs “*may be assessed more through expert opinion*”, whereas two participants mentioned that costs are not important in some emergencies. One participant was not sure whether costs should be included in RGs.

**Table 3:** Agreement level of participants to inclusion of costs, values and preferences, and external peer-review process in the development of Rapid Guidelines

Questions^a^	Median^b^	IQR^b^
Costs and resources	5	5 (2–6)
Patients values and preferences	6.5	6.5 (4.25–7)
External peer review	4.95	4.95 (3.5–6.0)

^a^ *See Appendix 1, for more information about the original question*

^b^*Agreement measured through a Likert scale from 1 to 7;* Strongly Agreed (7), Agreed (6), Somewhat Agreed (5), Neither Agreed or Disagreed (4), Somewhat Disagreed (3), or Disagreed (2), Strongly Disagreed (1)

In the follow-up question, five participants expanded on their thoughts about how patient’s values and preferences should be discussed or incorporated in RGs. Two participants provided suggestions on where information on patient’s values and preferences can be located, namely either through the involvement of non-governmental organisations, by examining the qualitative literature or conducting an ethnographic assessment. Two participants addressed the process for incorporating patient values and preferences; one stated that, for this step, there are “*no shortcuts* [in RGs] *compared to other guidelines*” and the other highlighted that they did not know how to assess patient’s values and preferences and that “*…it is difficult even for standard ones*”. Another participant stated that incorporating or discussing patient’s values and preferences was not typically time consuming. Finally, one participant stated that the inclusion of this information was “*fundamental*” to the context of the guideline.

Regarding external peer review, two participants stated that it could be skipped, whereas one clearly said it should not, and others state that finding good reviewers would be difficult.

“*If you are doing* [a rapid guideline] *for something really urgent, you will probably have all the best experts in the field, sitting in your guideline group, you will have difficulties finding other to do the peer-review; perhaps this step could be skipped*”

Four other participants addressed the process for conducting external peer review; three spoke of a less rigorous process than standard guidelines, with a turnaround of less than 2 days. One participant disagreed with the external review step arguing that the documents are “*comprehensive enough*”.

### Virtual meetings

All participants supported a combination of both virtual and face-to-face meetings. They identified facilitators and barriers to both approaches. Facilitators of virtual meetings included saving resources, efficiency, and “*…people may be less intimidated to give their opinion…*”. Barriers to virtual meetings included trying to gather everybody at the same time given other commitments and different time zones. Facilitators to holding face-to-face meetings included the ability to discuss more materials and to discuss controversial topics and reaching consensus. Four participants stated that “*face-to-face meetings are important*” and virtual meetings could complement them.

There were mixed suggestions for the number of face-to-face meetings (e.g. “*one face-to-face meeting is important*” or “*face-to-face meetings important in the beginning and in the end of the process*”).

“*I do think face-to-face* [meetings] *are still necessary. But probably in the beginning of the process an at the end of the process; in between, we can be more effective in doing virtual meetings*”

### Need for extra funding

Five participants said that the availability of more funding is critical for the development of guidelines, stating that additional funds would facilitate pooling data, hiring extra staff (i.e. people working full time), or hosting meetings. However, one participant recognised that additional funding might not facilitate the process, since many collaborators provide their time in-kind for the development of RGs. Two participants highlighted that the funds were not necessarily the barrier to developing RGs, but they could allow for faster diffusion of the recommendations. Creation of a specific team for development of RGs within the WHO was also recommended by one participant.

## Discussion

RGs are important for organisations tasked with providing prompt response and effective recommendations for emerging issues in health and disease in a variety of different settings. In this study, key participants from different areas of WHO involved in the development of RGs were interviewed to identify their experiences and perceptions about the RG development process.

### Strengths and weaknesses

The detailed qualitative methods, careful development of the interview process and interviews of real-life guideline developers at a high ranking international organisation by a team with a wealth of experience in guideline methodology represent strengths of this study. Given the limited information on this topic, the coupling of our systematic review with the analysis of existing RGs [10] lends support to the importance of our findings. Qualitative studies can reveal important information not captured in quantitative surveys. Furthermore, the lack of detailed guidance on RG development that we found in our systematic review in this series [10] indicates that these findings can facilitate the development of such guidance.

Among the limitations of this study, as in many qualitative studies, is the selection of key participants without considering representativeness. Conclusions drawn from the interviews do not necessarily represent the points of view of other members of the organisation nor the position of the organisation. Furthermore, we only interviewed participants from WHO.

### What are RGs? Situations requiring RGs and timing

The criteria for defining which situations will require RGs were not clear for participants. Some of them stated that it was necessary to describe which situations or scenarios could be considered a public health emergency within the WHO RG definition, and perhaps this could expedite the topic selection itself. The WHO Handbook utilised for this study suggested the development of RGs in response to a public health emergency, such as pandemic influenza, in which WHO is required to provide rapid global leadership and guidance. However, from the perspective of some participants, RGs may be developed in other situations, such as the management of multidrug-resistant organism infectious diseases or control of biological, chemical or radioactive hazards, among others. Other situations, such as diagnostic testing, new evidence on chronic diseases or post-traumatic disorders, were considered important by some participants, but not by others.

Additionally, as expected, the time required to develop RGs was a main concern and a central aspect for participants. RGs are typically required in epidemics or other situations in which there is pressure from countries to provide rapid guidance [15]. Therefore, most of the recommendations from participants about the process were somehow related to the possibility of reducing the time established in the WHO definition of RGs. A 1- to 3-month period was found adequate as long as there was flexibility according to the disease or emergency. Interestingly, the benefit of having flexibility was not only related to extending the duration but also to shortening the time to develop recommendations. A suggestion pointed to the development of so-called ‘interim’ or transitory recommendations within a week, in order to give the expected rapid guidance to the countries that need it, while the full RGs would be completed over a longer period. Other participants considered the timeframe suggested for the development of a rapid advice guideline not adequate for emergencies, on which guidance should be provided within a few days. The 2014 WHO Handbook for guideline development update now appropriately describes two types of guidelines in response to an emergency or urgent need: the ‘emergency (rapid response) guidelines’ and the ‘rapid advice guidelines’ [4]. The former will be developed for emergencies that require a response within hours to days; the recommendations may be performed based on previous guidelines or even on expert opinion alone. If the health emergency continues for an extended period, the initial emergency guideline must be reviewed and consider the evidence emerging from the event and a systematic review, resulting on the development of RGs. Furthermore, the handbook update considers the development of RGs for other situations, such as when a new drug becomes available, when new information on an existing technology is likely to change existing guidance, or because a Member State or external entity has made an urgent request for guidance. However, because of these reasons, the handbook recommends that the guideline may be developed with the same standards of other guidelines; a short timeframe could be achieved with allocation of appropriate resources [4].

In the same line, other authors have stated that rapid reviews can be seen as an ‘interim guidance’ while a formal systematic review in a second stage is developed [16, 17]. For instance, there are situations when immediate action, within hours or a day, is required such as in radiation or toxin exposures [15]. Thus, in emergency situations, a response may be performed between 1 and 3 months, while in cases where an interim guidance is to be given (i.e. and urgent response is needed), a recommendation during the first 1 to 2 weeks may be provided [15].

### Systematic reviews and evidence synthesis

We observed that some participants suggested following all steps expected in a regular guideline development process. Some participants did suggest avoiding certain steps of the process of regular guideline development but yet wanted to maintain a balance with rigor in the process. These participants suggested streamlining the evidence review and systematic review processes, reducing the peer-review process, and expediting the administrative issues related to the approval of RGs. On the other hand, few participants recommended skipping it and supporting decisions on experts’ opinion that would not describe the source of evidence for their opinion. Other participants suggested avoiding other steps but maintaining the systematic review. In this study, there was a perception that the systematic review of the evidence is a very time-consuming step in the RG development process. The methods for developing high-quality rapid reviews are a relatively new enterprise and can be challenging, but maintaining adherence to a minimum standard in the search, selection and synthesis of evidence methods, as well as maintaining standards of complete reporting, are key to avoiding the development of unreliable recommendations [18–20].

In fact, rapidly developed guidelines should not be associated with lack of trustworthiness. We believe that adequate planning which is supported by existing guidelines for guidelines [4] can result in the development of a trustworthy RG, without eliminating the systematic review required to retain evidence-based principles. In contradiction to these principles, some participants perceived experts as a very reliable source of evidence, stating that they can bring the best evidence. Furthermore, two participants suggested that methodologists may be a ‘barrier’ when there is a need for very rapid response. We think this may be because methodologists are associated with a long systematic process while expert opinions can be related to expedited decision-making processes. In contrast, other authors suggest that methodologists, as experts of the guideline development process, are critical members in the process of any evidence-based guideline to ensure transparency [21]. Striking the right balance between obtaining information and avoiding bias is challenging. There is no reason to believe that a rapid process solely relying on expert opinion would avoid the concerns previously identified with regular guidelines at WHO [6]. In fact, conducting rapid reviews was suggested to overcome part of this challenge. In addition, we previously described that, in addition to rapid reviews, transparent methods for evidence assessment can be used for emergent and urgent situations [16, 17]. However, we believe that these efforts require methodological expertise. If methodologists facilitate the adherence to these principles of high rigor while recognising time constraints they would no longer be perceived as barriers, but rather as facilitators of systematic and adaptable processes.

### Panel composition

Finally, when participants were asked about the Influenza RG process, they also recommended the involvement of members of the WHO GRC in regular meetings during the RG process. Interestingly, this recommendation arose at the time of data collection although this committee did not exist at WHO at the time of the Influenza RG development. Nevertheless, this idea highlighted the need for appropriate planning and involvement of key actors within the organisation to enhance the process and avoid administrative delays after RGs have been drafted.

### External review, values and preferences, and external review

Perceptions about the importance of including an external review process varied. The external review or peer-review processes are an important step in any scientific product (including practice guidelines) and are inherent in a guideline development process. Other authors suggest that external review increases quality, rigor and trustworthiness of guidelines, as recommended by guideline assessment instruments such as the Appraisal of Guidelines for Research & Evaluation Instrument (AGREE II) [22] used by guideline-development organisations [1–4, 23]. The WHO Manual for Developing Guidelines [4] states that, in RGs, peer review can be limited to review of the complete draft only, immediately before final clearance, perhaps by 3–6 experts [4]. Efforts to maintain this process while avoiding unwanted delays should be made. One strategy may be identifying external peer reviewers not involved in the process from the beginning. Participants’ criticism of the peer-review step was deemed as having the potential to impact the final recommendations. In fact, recommendations should preferably not change because of peer review as it would require reconvening of the panel.

Finally, consideration of costs and resources and of patients’ values and preferences when developing RGs was seen predominantly, but not consistently, as important. Concerns about the importance of costs on emergency situations were raised, as well as their importance when implementing the guidelines. Furthermore, there was some agreement in the inclusion of values and preferences in the process, which was considered an action that can be easily implemented in the process.

### RG development process and funding

All participants supported the process with mixed meetings (face-to-face and virtual) despite both types being associated with advantages and disadvantages. Careful planning of the process with one or two meetings might lead to efficient use of time while allowing appropriate deliberation and discussion. Increased funding was considered as a way to improve the process by most of participants. This action was also seen as a potential enabler of human resource working allowing the simultaneous work of teams of experts and methodologists, which would result in a more efficient process.

## Conclusions

RGs are an important tool to inform decision-making in the context of health emergencies or urgencies as they can allow for evidence-based recommendations. Our study identified appropriate balance between the rigor and speed of the process as key themes. While systematic reviews are the basis of any guideline process, some participants considered them to be an obstacle. This can be overcome by conducting rapid reviews. Peer-review processes were identified as a major issue in providing rapid guidance. While our work is based on interviews at WHO, they might be useful to other organisations dealing with emergencies or urgencies. Based on the findings of this and the prior article [10], and utilising the GIN-McMaster Checklist for Guideline Development, the next and final article in this series will provide guiding principles for the development of RGs [24].

## Additional files

Additional file1: Interview questions. (DOCX 19 kb)

Additional file 2: WHO rapid Advice Guideline definition. (DOCX 18 kb)

Additional file 3: Avian influenza rapid advice guideline – timeline. (DOCX 20 kb)

## Abbreviations

GRC: Guideline Review Committee
RGs: rapid guidelines
