# Supplementary material for: Development of rapid guidelines: 2. A qualitative study with WHO guideline developers
Source: Health Res Policy Syst. 2018 Jul 13;16:62. doi: 10.1186/s12961-018-0329-6 (PMC6044000; doi:10.1186/s12961-018-0329-6)
Supplement: Supplementary file 1 — Interview questions. (DOCX 19 kb) [file 12961_2018_329_MOESM1_ESM.docx]

# Additional file 1. Interview questions

| **-Please review the WHO rapid advice guideline definition and respond (questions 1 and 2)**  1 – What are your thoughts about the definition of the WHO for rapid advice guidelines?  2. Beyond this definition, can you think of circumstances or examples where a rapid advice guideline should be performed instead of a standard guideline?  **The following questions are related in general to the RG processes (questions 3-17)**  3. Considering the scenarios below, to what extent do you agree or disagree that a RG should be performed? **(Closed-ended question, Scenarios to evaluate the agreement with the Likert Scale- See Table 3 in manuscript)**  4. How feasible, in general, is the development of RGs and what are the main obstacles in the development of RGs?  5. Considering the whole process (from the idea conception to final publication on the WHO website), what is an acceptable timeline for a rapid advice guideline?  6. In your opinion, what are the most time-consuming aspects, and steps, when developing a guideline?  7. Ideally, guidelines should be fully evidence-based. Usually, full systematic reviews are conducted for each question that is drafted for the guideline in order to provide the best evidence available. However, this is one of the most time consuming steps in the development process. In your opinion, how should the evidence be compiled for RGs?  8. Considering the time spent on this step, to what extent do you agree or disagree that costs and resource should be discussed and incorporated in a rapid advice guideline? **(Closed-ended question, Likert Scale)**  9. Do you have any thoughts or suggestions about how costs and resource considerations should be discussed and incorporated in a RG?  10 - Considering the time spent on this step, to what extent do you agree or disagree that patient values and preferences, should be discussed and incorporated in a rapid advice guideline? **(Closed-ended question, Likert Scale)**  11 – Do you have any thoughts or suggestions about how patient values and preferences should be discussed and incorporated in a rapid advice guideline?  12 - Considering the time spent in this step, to what extent do you agree or disagree that external peer review should be done for a rapid advice guideline? **(Closed-ended question, Likert Scale)**  13 - What could make the external review process faster? Should external peer review be omitted for RGs?  14 – There are some debates about virtual meetings replacing face-to-face meetings (e.g. skype). What kind of approach do you prefer and why?  15 – Do you think that having more funding available for the development of the guideline would make the process faster, and how would you use the extra funding?  16 – Regarding the panel composition (guideline panel members), who would you consider to not involve in order to make the process faster?  17 – How do you think this timeline could be improved?  **-Please review this timeline for the WHO rapid advice guideline for avian influenza**  18 – Do you think this timeline is feasible and applicable to most rapid advice guidelines that would be produced in your area of work? |
| --- |
