# Supplementary material for: Development of rapid guidelines: 2. A qualitative study with WHO guideline developers
Source: Health Res Policy Syst. 2018 Jul 13;16:62. doi: 10.1186/s12961-018-0329-6 (PMC6044000; doi:10.1186/s12961-018-0329-6)
Supplement: Supplementary file 2 — WHO rapid Advice Guideline definition. (DOCX 18 kb) [file 12961_2018_329_MOESM2_ESM.docx]

# Additional file 2. WHO rapid Advice Guideline definition

| **Definition and indication for Rapid advice guidelines***  *“A rapid advice guideline is produced in response to a public health emergency (such as pandemic influenza), in which WHO is required to provide rapid global leadership and guidance. This type of document needs to be produced in a time span of 1–3 months and will be evidence-informed, but it may not be supported by full systematic reviews of the evidence. It will be prepared mainly by the responsible WHO staff members with external consultation and peer review. It must be published with a “review-by” date that indicates when the guidance will become invalid, or when it will be updated or converted to a standard guideline.”* |
| --- |

*Source: WHO handbook for guideline development, first edition, 2011.
