# Supplementary material for: Development of rapid guidelines: 2. A qualitative study with WHO guideline developers
Source: Health Res Policy Syst. 2018 Jul 13;16:62. doi: 10.1186/s12961-018-0329-6 (PMC6044000; doi:10.1186/s12961-018-0329-6)
Supplement: Supplementary file 3 — Avian influenza rapid advice guideline – timeline. (DOCX 20 kb) [file 12961_2018_329_MOESM3_ESM.docx]

# Additional file 3. (Avian influenza rapid advice guideline – timeline)

| **Key Steps in the Development of WHO Rapid Advice Guidelines** | |
| --- | --- |
| **Decision about the topic and focus of the guidelines**  **Decision about group composition and invitation of panel**  **Formulation of questions and rating the importance of outcomes** | **January 2006** |
| **Literature search and preparation of evidence profiles**  WHO panel co-chair met with systematic reviewers Panel chair and WHO panel co-chair corresponded electronically with systematic reviewers  **Review of evidence profiles and draft guidelines**  Panel chair met with WHO panel co-chair and systematic reviewers | **February 17, 2006** |
| **Panel meeting**  Information about methods and agreement on procedures at the meeting  Declaration of conflicts of interest  Deliberation regarding the balance of benefits, harms, and costs for each question  Agreement on recommendations, including the strength of recommendations, and research priorities  Plans for updating the guidelines | **March 28–29, 2006** |
| **Agreement on final text of guidelines**  Circulation of draft guidelines  Approval by panel members | **April 21, 2006** |
| **Approval/publication by WHO** | **May 19, 2006** |

*Source: Schunemann HJ, Hill SR, Kakad M, Bellamy R, Uyeki TM, Hayden FG, et al. WHO Rapid Advice Guidelines for pharmacological management of sporadic human infection with avian influenza A (H5N1) virus. The Lancet Infectious diseases. 2007;7(1):21-31 (7)
